# A retrospective study on IVF outcome in euthyroid patients with anti-thyroid antibodies: effects of levothyroxine, acetyl-salicylic acid and prednisolone adjuvant treatments

**DOI:** 10.1186/1477-7827-7-137

**Published:** 2009-11-27

**Authors:** Alberto Revelli, Simona Casano, Luisa Delle Piane, Giuseppina Grassi, Gianluca Gennarelli, Daniela Guidetti, Marco Massobrio

**Affiliations:** 1 Reproductive Medicine and IVF Unit, Department of Obstetrical and Gynecological Sciences, University of Torino, OIRM-S. Anna Hospital, Torino, Italy; 2 LIVET Infertility and IVF Clinic, Torino, Italy

## Abstract

**Background:**

Anti-thyroid antibodies (ATA), even if not associated with thyroid dysfunction, are suspected to cause poorer outcome of in vitro fertilization (IVF).

**Methods:**

We retrospectively analyzed: (a) the prevalence of ATA in euthyroid infertile women, (b) IVF outcome in euthyroid, ATA+ patients, and (c) the effect of adjuvant treatments (levothyroxine alone or associated with acetylsalicylic acid and prednisolone) on IVF results in ATA+ patients. One hundred twenty-nine euthyroid, ATA+ women undergoing IVF were compared with 200 matched, ATA-controls. During IVF cycle, 38 ATA+ patients did not take any adjuvant treatment, 55 received levothyroxin (LT), and 38 received LT +acetylsalicylic acid (ASA)+prednisolone (P).

**Results:**

The prevalence of ATA among euthyroid, infertile patients was 10.5%, similar to the one reported in euthyroid women between 18 and 45 years. ATA+ patients who did not receive any adjuvant treatment showed significantly poorer ovarian responsiveness to stimulation and IVF results than controls. ATA+ patients receiving LT responded better to ovarian stimulation, but had IVF results as poor as untreated ATA+ women. Patients receiving LT+ASA+P had significantly higher pregnancy and implantation rates than untreated ATA+ patients (PR/ET 25.6% and IR 17.7% vs. PR/ET 7.5% and IR 4.7%, respectively), and overall IVF results comparable to patients without ATA (PR/ET 32.8% and IR 19%).

**Conclusion:**

These observations suggest that euthyroid ATA+ patients undergoing IVF could have better outcome if given LT+ASA+P as adjuvant treatment. This hypothesis must be verified in further randomized, prospective studies.

## Background

Autoimmune thyroid diseases are rather frequent in women in the childbearing age, affecting 5-20% of them [1]. They are characterized by the presence of anti-thyroglobulin and anti-thyroperoxidase antibodies, grouped under the definition of anti-thyroid antibodies (ATA). ATA are often detected in subjects complaining of hypo- or hyperthyroidism, but are not rarely found in patients without any sign of thyroid dysfunction [1].

Some evidence suggest that ATA could exert a negative influence on the female reproductive potential. Women with no signs of thyroid dysfunction who were ATA+ risk spontaneous miscarriage three- to five-folds more than ATA-women [2]. Moreover, some studies reported a significantly higher ATA prevalence in subfertile women complaining of pelvic endometriosis [3,4], premature ovarian failure (POF) [5,6], polycystic ovary (PCO) [7], or hyperprolactinemia [8].

Some authors even reported an unexpectedly high ATA prevalence in euthyroid women with a history of three or more unsuccessful IVF cycles [9], and ATA-positivity was found to be associated with a low pregnancy rate in IVF [10-12]. On the contrary, other studies failed to detect any difference in IVF success rate between ATA+ and ATA-women [13-15].

In our study, we analyzed the prevalence of ATA in infertile women and compared it with that observed in age-matched, fertile controls. Further, we retrospectively analyzed IVF outcome in euthyroid ATA+ women and compared it with ATA-controls. Differently from previous reports, we also studied the effect of some adjuvant medical treatments that are frequently given to ATA+ women during IVF (levothyroxine alone or associated with acetyl-salicylic acid and prednisolone), on IVF results. Levothyroxine (LT), in fact, is claimed to reduce ATA level [16] and lower the risk of miscarriage in ATA+ women [17], whereas acetyl-salicylic acid (ASA) plus prednisolone (P) was reported to improve IVF outcome in women with autoimmune disorders [18-21]. Aim of the present study is to check the effectiveness of such adjuvant treatments as tools to improve IVF outcome in ATA+ patients.

## Methods

### Patients

Our retrospective analysis included 3076 infertile women referring to the IVF Unit between February 2004 and May 2008. The diagnostic workout included anti-thyroglobulin and anti-thyroperoxidase antibodies (ATA) detection, as well as the assessment of circulating TSH, f-T_4 _and f-T_3 _levels. According to our guidelines, a woman was considered ATA+ when the antibody level reached 40 UI/mL for anti-thyroglobulin and 35 UI/mL for anti-thyroperoxidase antibodies respectively; patients with lower levels were considered ATA-.

Among all patients, 42 resulted to be affected by hypo- or hyperthyroidism and were excluded from the study; 3034 women were euthyroid, and among them 319 were ATA+. One hundred twenty-nine euthyroid, ATA+ women entered the IVF program and were further subdivided into three subgroups: a) those who were not taking any adjuvant medication at the time IVF (group A, n = 38), b) those who despite being euthyroid received levothyroxine (LT, 50 mcg/d) as adjuvant treatment during IVF (group B, n = 55), and c) those who despite being euthyroid received LT, acetyl-salicylic acid (ASA) and prednisolone (P) as adjuvant treatment during IVF (group C, n = 36). Adjuvant treatments were prescribed by different endocrinologists taking care of the patients' thyroid conditions (not belonging to our team) without any known selection criteria apart from their personal, clinical experience. As controls, we considered 200 euthyroid, ATA- patients who underwent IVF in the same period and had oocyte retrieval the same day of the ATA+ women (group D).

The 329 patients (129 cases and 200 controls) included in the study underwent 352 IVF cycles (52 in group A, 56 in group B, 44 in group C, 200 in group D) and obtained 95 pregnancies, with an overall pregnancy rate/cycle of 27%. Sixteen pregnancies subsequently aborted, and the overall abortion rate was 16.8%.

### ATA assay

Anti-thyroglobulin and anti-thyroperoxidase antibodies were measured by Pharmacia Diagnostic commercial kits (Pharmacia, Sweden) using an immunofluorescence assay, with normal values set at < 40 UI/ml (anti-thyroglobulin) and < 35 U/ml (anti-thyroperoxidase).

### IVF procedure

Superovulation was induced using a standard "long" protocol with GnRH agonist (Buserelin) plus gonadotropins (rFSH or hMG) at appropriate doses (100-450 IU), that were estimated according to the woman's age, antral follicle count and basal (day 3) FSH. Ovarian response to gonadotropins was monitored by transvaginal ultrasound plus serum E_2 _measurement every third day from day 7 of stimulation. The final follicle maturation was triggered by giving 10,000 IU hCG i.m or s.c. when the leading follicle reached 18 mm, with appropriate serum E_2 _levels.

Transvaginal ultrasound-guided oocyte aspiration (OPU) was performed approximately 36 hrs after hCG injection under local anaesthesia (paracervical block). Either IVF or ICSI were performed according to the clinical indication. According to Italian legal rules, a maximum number of three oocytes were inseminated and unselected embryos were transferred into the uterus (ET) after 48 hours of in vitro culture. The luteal phase was supplemented by vaginally administered natural progesterone (400 mg/d). After 14 days from ET, serum hCG was assayed and pregnancy was eventually confirmed by transvaginal ultrasound.

### Adjuvant medical treatments

Patients in groups A (ATA+, no adjuvant treatment) and D (ATA-) received a standard IVF treatment as described above, without any adjuvant medication.

ATA+ patients treated with LT (group B) received LT 50 mcg/d orally throughout the whole IVF cycle, from the first day of stimulation to the day of the pregnancy test. Patients who had a positive pregnancy test and a clinically confirmed pregnancy continued LT until 10 weeks of gestational age. After this time, the option to continue LT treatment was left to doctors taking care of the pregnancy.

ATA+ patients treated with LT, ASA and P (group C) received a daily dose of 100 mg orally administered ASA + 10 mg P from the first day of stimulation; P was increased to 30 mg/d for 5 days starting the day of ET, and subsequently returned to 10 mg/d until the day of hCG test. This ASA+P protocol was previously published by our group and proven to be safe in IVF patients [22]. Patients of group C with a positive pregnancy test and a clinically confirmed pregnancy continued the LT+ASA+P regimen until 10 weeks of gestational age. After this time, the option to continue the treatment was left to doctors taking care of the pregnancy.

### Statistics

The following variables were recorded: stimulation length, total gonadotropin dose, number of retrieved oocytes, ovarian responsiveness to stimulation (gonadotropin dose/retrieved oocyte), fertilization rate, number of transferred embryos/cycle, pregnancy rate (PR)/started cycle, PR/OPU, PR/ET, abortion rate, ongoing pregnancy rate/ET.

The comparison among groups was performed using JMP software [23]. Since variables were found to be normally distributed at Lilliefors test, the ANOVA test, χ^2 ^test and Fisher's exact test were used.

## Results

### Prevalence of ATA in euthyroid, infertile women

The prevalence of ATA among euthyroid, infertile patients was 10.5% (319/3034). Patients with pelvic endometriosis or reduced ovarian reserve had significantly higher ATA prevalence (21.8% and 22.5%, respectively, p < 0.01) if compared to women with tubal disease (5.6%) or belonging to couples with idiopathic infertility (13.9%) or male-related infertility (6.4%).

The prevalence of PCO was similar in ATA+ and ATA- patients (13.5% vs. 19.3%, respectively, p = ns). Hyperprolactinemia was significantly more frequent among ATA+ women (14.1% vs. 7.2%, p < 0.01).

### ATA and IVF outcome

ATA+ and ATA- patients undergoing IVF had similar age, BMI, PCO prevalence, smoke habit prevalence, basal FSH levels, antral follicle count, TSH and fT4 levels (Table 1).

**Table 1:** Clinical characteristics of the patients included in the study

	Group A	Group B	Group C	Group D	p
*Patients*	38	55	36	200	ns
*Age (yrs)*	37.0 +/- 3.5	35.1 +/- 4.1	35.7 +/- 3.8	36.6 +/- 3.6	ns
*BMI*	22.1 +/- 3.7	22.6 +/- 3.5	22.8 +/- 2.8	21.8 +/- 3.0	ns
*PCO (%)*	8.6	10.9	11	10.8	ns
*Smoke (%)*	10.5	9	8.5	12.3	Ns
*FSH (UI/L)*	7.3 +/- 2.2	7.3 +/- 2.5	6.7 +/- 1.8	7.1 +/- 2.5	Ns
*Antral follicle count*	5.8 +/- 3.1	6.7 +/- 3.4	6.9 +/- 3.5	7.0 +/- 1.9	Ns
*TSH (mIU/L)*	2.0 +/- 1.2	2.1 +/- 1.3	2.1 +/- 1.5	2.2 +/- 1.1	Ns
*fT4 (pg/mL)*	10.6 +/- 2.8	9.9 +/- 3.5	10.1 +/- 3.2	10.3 +/- 3.0	Ns
*fT3 (pg/mL)*	3.1 +/- 1.4	3.0 +/- 1.5	3.2 +/- 1.7	3.3 +/- 1.9	Ns

Group A: ATA+, untreated patients. Group B: ATA+ patients treated with LT adjuvant therapy. Group C: ATA+ patients treated with LT+ASA+P adjuvant therapy; Group D: ATA-controls.

ATA+ patients receiving no adjuvant medical treatment (group A) showed a significantly worse ovarian responsiveness to gonadotropins than ATA-women (group D), needed a higher gonadotropin dose, had longer stimulation times and finally retrieved less oocytes (Table 2). Oocyte and embryo quality was worse in group A than in group D, the former obtaining lower implantation, pregnancy and ongoing pregnancy rates (Table 2).

**Table 2:** IVF outcome of the patients included in the study

	Group A	Group B	Group C	Group D
*IVF cycles*	52	56	44	200
*Total Gn dose (IU)*	3714 +/- 1499 *c*	3430 +/- 1722	3000 +/- 1358 *b*	2755 +/- 1216 *a*
*Stimulation length (d)*	12.3 +/- 2.1	12.2 +/- 2.5	11.4 +/- 1.6 *b*	11.5 +/- 1.8 *b*
*OPU*	50	55	44	200
*Retrieved oocytes*	6.5 +/- 4.3	7.9 +/- 5.4	10.7 +/- 6.4 *b*	8.7 +/- 5.2 *b*
*Gn dose/oocyte (IU)*	1028 +/- 1141	878 +/- 1045	448 +/- 520 *b*	539 +/- 796 *b*
*Fertilization rate (%)*	74.2	78.5	82.5	83.0
*ET*	40	45	39	177
*Pregnancies*	4	11	14	66
*PR/started cycle (%)*	7.7 *c*	19.6	31.8 *b*	33.0 *b*
*PR/OPU (%)*	8.0 *c*	20.0	31.8 *b*	33.3 *b*
*PR/ET (%)*	10.0 *c*	24.4	35.9 *b*	37.3 *b*
*Implantation rate (%)*	4.7 *c*	14.4	17.7 *b*	19.0 *a*
*Abortions*	1	3	4	8
*Abortion rate (%)*	25.0	27.3	28.5	12.0 *a*
*Ongoing PR/OPU (%)*	6.0 *c*	14.5	22.7 *b*	29.3 *b*
*Ongoing PR/ET (%)*	7.5 *c*	17.8	25.6 *b*	32.8 *b*

Group A: ATA+, untreated patients. Group B: ATA+ patients treated with LT adjuvant therapy. Group C: ATA+ patients treated with LT+ASA+P adjuvant therapy; Group D: ATA-controls.

*a *= p < 0.01 vs groups A, B and C

*b *= p < 0.01 vs groups A, and B

*c *= p < 0.01 vs group B and C

ATA+ women treated with adjuvant LT (group B) showed a slightly better ovarian responsiveness to gonadotropins, needed a lower gonadotropin dose and obtained more oocytes at OPU than untreated patients (group A) (Table 2). Anyway, their ovarian responsiveness to stimulation was significantly poorer if compared to ATA-controls (Table 2). The pregnancy and implantation rates were significantly higher in group B than in group A, but still significantly lower than in group D (Table 2).

The adjuvant treatment with LT+ASA+P (group C) was associated with a significantly higher ovarian responsiveness to stimulation than the one observed with other ATA+ patients (groups A and B)(Table 2). Actually, it was similar to the one of ATA-controls (Table 2). The pregnancy, implantation and ongoing pregnancy rates in group C were comparable to ATA- patients, and significantly higher than those observed in groups A and B (Table 2).

Interestingly, the abortion rate was significantly higher in all ATA+ patients, and was unaffected by adjuvant treatments (Table 2).

## Discussion

Anti-thyroglobulin, anti-thyroperoxidase antibodies, or both, are frequently detected in subjects with hypo- or hyperthyroidism of autoimmune origin, and can also be found in patients without any sign of thyroid disease. According to some reports, ATA positivity in euthyroid women could be linked to reproductive problems, such as increased abortion rate or infertility [2].

In the population of euthyroid, infertile women that we observed, the prevalence of ATA was 10.5%, comparable to what reported in large population studies on euthyroid, fertile women between 18 and 45 years (13.8%; 95% CI 7.6-19.9) [24]. Our observation, therefore, does not support the concept of a strong association between ATA posotivity and female infertility. It must be remarked, however, that we considered clinically relevant (potentially able to affect fertility) only ATA levels above a given threshold, whereas some reports on fertile women included all levels of positivity.

We observed that some specific causes of female subfertility, such as pelvic endometriosis or reduced ovarian reserve, are more frequently associated with ATA than other infertility factors. The association between ATA and pelvic endometriosis, indeed, was already reported [3,4]; it could be based on a common dysfunction of the immune system, as there is abundant evidence of the link between endometriosis and specific immunological features [25]. Also premature ovarian failure (POF), that is considerable an extreme form of reduced ovarian reserve, is often associated with autoimmune disorders [26], and anti-zona pellucida antibodies able to cross-react with thyroid antigens have been detected in POF patients: they could represent a link between POF syndrome and anti-thyroid autoimmunity [27]. We also observed, in agreement with other reports [8], an increased prevalence of ATA in hyperprolactinemic women. PRL is known to exert immunomodulatory effects that could lead to increased autoreactivity of the immune system [28]. Differently, we failed to confirm the previously reported observation [7] of increased PCO prevalence among ATA+ women.

Observing IVF outcome, we noticed that it tended to be noticeably poorer in ATA+ than ATA-women. ATA+ patients who did not take any adjuvant treatment responded significantly worse to ovarian stimulation than ATA-controls, their gonadotropin dose/retrieved oocyte ratio (a reliable marker of ovarian responsiveness to stimulation) being significantly higher. A normal responsiveness to ovarian stimulation in ATA+ women was previously reported [29], but that study included only 16 ATA+ patients and unfortunately did not report their basal FSH level, nor their antral follicle count, two variables that deeply influence ovarian response to hormones.

We observed that the presence of ATA could have affected also the oocyte and consequently the embryo deriving from it; in fact, we recorded a lower fertilization rate, pregnancy and implantation rates in untreated ATA+ patients vs. ATA-controls. Interestingly enough, Bussen found a higher prevalence of ATA in infertile women with a history of three or more unsuccessful IVF attempts [9], and other studies reported poorer IVF results in ATA+ patients [10-12]. Other authors, however, failed to confirm the existence of an association between ATA and IVF failure [13-15]. The inconsistency in the reported results could be linked to several factors, like different criteria of patient inclusion in the study (e.g. only those with unexplained infertility or all infertile patients), different ATA level threshold needed to include a patient, different criteria to chose the control group, etc.

We observed a significantly higher abortion rate after IVF in ATA+ patients vs. ATA-controls. A similar finding was already reported by others [10,14,15,30] and it is not surprising since an increased abortion rate occurs in ATA+ women even after spontaneous conception [2].

ATA+ women who despite being euthyroid received a low dose of LT during IVF showed a slightly higher ovarian responsiveness to gonadotropins, but IVF outcome was still significantly poorer in comparison to ATA- patients. Some doctors use to give a low dose of LT to ATA-positive euthyroid women because this treatment was reported to reduce ATA level [16] and to lower the risk of miscarriage in recurrent aborters who conceived spontaneously [17]. In our study, the abortion rate after IVF was not influenced by LT, and IVF results were not relevantly improved; this could be due to the high estradiol levels reached during IVF (approximately ten folds higher than in the physiological cycle) that could have induced high levels of thyroxine binding globulin, in turn able to bind and partially inactivate LT.

The adjuvant treatment with acetylsalicylic acid (ASA) combined with prednisolone (P) is advised by some doctors to patients with autoantibodies undergoing IVF; in fact, it was reported to improve IVF outcome in women with autoimmune disorders [18-21] or with recurrent abortions [31]. Beneficial effects of this adjuvant treatment on IVF outcome, however, were not confirmed when patients without autoantibodies were studied [22,32]. Our retrospective analysis shows that women treated with LT+ASA+P showed a higher ovarian responsiveness to gonadotropins and higher pregnancy, implantation and ongoing pregnancy rates than other ATA+ patients. Their IVF results were comparable to those observed in ATA- subjects. LT+ASA+P treatment, however, was unable to decrease the abortion rate, that remained significantly higher than the one observed in women without ATA. The advantage of giving LT+ASA+P to euthyroid ATA+ women could depend on the combination of the immunomodulatory activity of LT and P with a better ovarian and endometrial blood perfusion due to ASA [33]. Prednisolone could also lower the local inflammatory reaction to the ET catheter and facilitate implantation through improving the tolerance of the immune system toward the transferred embryos [34].

In conclusion, this retrospective study on euthyroid ATA+ women shows that: a) ATA-positivity in infertile women is as frequent as in euthyroid fertile women of comparable age; only in case of pelvic endometriosis, reduced ovarian reserve, or hyperprolactinemia, ATA prevalence is significantly higher than normal; b) ATA+ patients have significantly worse IVF outcome when compared to ATA-women; c) the adjuvant treatment with LT gives a slight improvement of ovarian responsiveness to stimulation, but has no benefit on IVF results; d) the adjuvant treatment with LT+ASA+P significantly improves IVF outcome; e) no adjuvant treatment decreases the abortion rate after IVF.

The beneficial effect of LT+ASA+P adjuvant treatment on IVF outcome observed herein in euthyroid, ATA+ patients, together with its low potential risk [22], suggests its possible clinical, targeted application in this specific subgroup of IVF patients. However, our findings need to be validated in prospective randomized investigations before the application of LT+ASA+P adjuvant treatment is encouraged.

## Competing interests

The authors declare that they have no competing interests.

## Authors' contributions

AR, SC, LDP, DG and GG participated in data collection and manuscript writing. GG performed the statistical analysis. MM critically reviewed the manuscript. All authors read and approved the final manuscript.
